# ENU-induced phenovariance in mice: inferences from 587 mutations

**DOI:** 10.1186/1756-0500-5-577

**Published:** 2012-10-24

**Authors:** Carrie N Arnold, Michael J Barnes, Michael Berger, Amanda L Blasius, Katharina Brandl, Ben Croker, Karine Crozat, Xin Du, Celine Eidenschenk, Philippe Georgel, Kasper Hoebe, Hua Huang, Zhengfan Jiang, Philippe Krebs, Diantha La Vine, Xiaohong Li, Stephen Lyon, Eva Marie Y Moresco, Anne R Murray, Daniel L Popkin, Sophie Rutschmann, Owen M Siggs, Nora G Smart, Lei Sun, Koichi Tabeta, Victoria Webster, Wataru Tomisato, Sungyong Won, Yu Xia, Nengming Xiao, Bruce Beutler

**Affiliations:** 1 Department of Genetics, The Scripps Research Institute, La Jolla, CA, 92037, USA; 2 Center for Genetics of Host Defense, UT Southwestern Medical Center, 5323 Harry Hines Boulevard, 8505, Suite NB9-202D, Dallas, TX, 75390, USA

**Keywords:** N-ethyl-N-nitrosourea, Mouse, C57BL/6J, Mutagenesis, Genetic screen, PolyPhen-2, Strand asymmetry, Phenotype

## Abstract

**Background:**

We present a compendium of *N*-ethyl-*N*-nitrosourea (ENU)-induced mouse mutations, identified in our laboratory over a period of 10 years either on the basis of phenotype or whole genome and/or whole exome sequencing, and archived in the Mutagenetix database. Our purpose is threefold: 1) to formally describe many point mutations, including those that were not previously disclosed in peer-reviewed publications; 2) to assess the characteristics of these mutations; and 3) to estimate the likelihood that a missense mutation induced by ENU will create a detectable phenotype.

**Findings:**

In the context of an ENU mutagenesis program for C57BL/6J mice, a total of 185 phenotypes were tracked to mutations in 129 genes. In addition, 402 incidental mutations were identified and predicted to affect 390 genes. As previously reported, ENU shows strand asymmetry in its induction of mutations, particularly favoring T to A rather than A to T in the sense strand of coding regions and splice junctions. Some amino acid substitutions are far more likely to be damaging than others, and some are far more likely to be observed. Indeed, from among a total of 494 non-synonymous coding mutations, ENU was observed to create only 114 of the 182 possible amino acid substitutions that single base changes can achieve. Based on differences in overt null allele frequencies observed in phenotypic vs. non-phenotypic mutation sets, we infer that ENU-induced missense mutations create detectable phenotype only about 1 in 4.7 times. While the remaining mutations may not be functionally neutral, they are, on average, beneath the limits of detection of the phenotypic assays we applied.

**Conclusions:**

Collectively, these mutations add to our understanding of the chemical specificity of ENU, the types of amino acid substitutions it creates, and its efficiency in causing phenovariance. Our data support the validity of computational algorithms for the prediction of damage caused by amino acid substitutions, and may lead to refined predictions as to whether specific amino acid changes are responsible for observed phenotypes. These data form the basis for closer in silico estimations of the number of genes mutated to a state of phenovariance by ENU within a population of G3 mice.

## Findings

### Background

*N*-ethyl-*N*-nitrosourea (ENU) is a germline mutagen that transfers its ethyl group to a nucleophilic nitrogen or oxygen in nucleic acids [1-5]. These transferred ethyl groups form DNA adducts that cause mispairing and base-pair substitutions [4], which are transmitted from spermatogonial stem cells to spermatids and finally sperm [5].

Most of the mutations caused by ENU are single base-pair substitutions (*e.g.* A/T to T/A transversions (44%) or A/T to G/C transitions (38%)) [2,4,5]. When they fall within coding regions, these mutations cause missense (64%), splicing (26%), nonsense (10%), or make-sense (*i.e.* a stop codon is converted back to an amino-acid-coding codon) (~1%) mutations [4-6]. ENU can also disrupt normal splicing, usually by changing nucleotides that fall within introns, and occasionally by changing nucleotides within coding region as well; *i.e.*, by creating novel splice sites.

Analysis of ENU-induced mutations revealed that ENU action was more biased towards genes with higher G + C content, while mutated nucleotides were more frequently flanked by G or C [7]. As a result of this bias, it is probable that amino acid changes specified by A/T to T/A or A/T to G/C mutations would be affected more readily than those that require G/C to C/G or G/C to T/A changes [5]. ENU-induced mutations have been predicted to occur at a frequency of one mutation per every 1 to 2.7 Mbp of genomic DNA, depending upon strain and dosage [5,8-10]. Still higher estimates of mutation rate have recently been made, based on whole exome sequencing carried out using the DNA of G1 mice [11].

Considering the size of the mouse haploid genome and accepting a rate of 1 mutation per Mbp of DNA, it is estimated that each ENU-treated male (G0) gamete would carry ~3000 mutations. It is further estimated that ~30 of these mutations would result in coding changes in G1 animals [5,12]. And the preponderance of phenotype caused by ENU results from coding change, despite rare examples to the contrary [13,14].

Here, we summarize mutations created using ENU mutagenesis in our own laboratory and publicly described during the past 10 years. Using both positional cloning and massively parallel short read sequencing, we have acquired a collection of mutations that clearly cause phenotype, and also a collection of mutations that may do so, but are not known to. These data permit inferences concerning the frequency at which ENU-induced missense errors will disrupt protein structure so as to cause a detectable phenotype. We confirm that A→T transversions and A→G transitions are the most common substitutions induced by ENU. In addition, we confirm that ENU shows marked asymmetry in its effects on sense vs. antisense strands. Of particular note, we estimate the likelihood that an ENU-induced missense mutation will, in the general case, cause damage of a degree sufficient to elicit detectable phenovariance.

### ENU mutagenesis program and phenotypic screens

**Figure 1:**
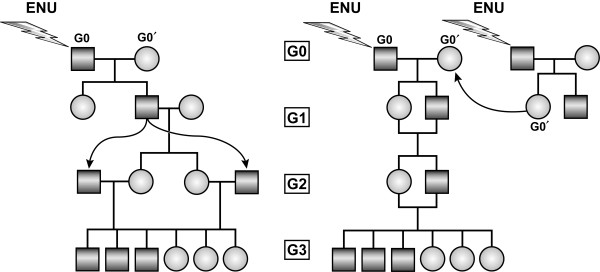
**Inbreeding strategies for generating G3 mice carrying homozygous ENU-induced mutations.** (Left) The G0 male is mutagenized; the G0’ female is a wild type C57BL/6J animal. G1 males are mated to wild type C57BL/6J females, and the resulting G2 daughters are backcrossed to their G1 father to yield G3 mice. (Right) The G0 male is mutagenized; the G0 female (the daughter of another mutagenized male and a wild type C57BL/6J female) also carries ENU-induced mutations. G1 siblings are intercrossed to obtain G2 mice. G3 mice are obtained from intercrosses of G2 siblings.

Between 2001 and 2011, an ENU mutagenesis program was operated by the Beutler laboratory at The Scripps Research Institute in La Jolla, CA. A total of 38248 G1 and 113816 G3 mutant mice were generated and weaned during those years, the latter resulting partly from a backcross breeding strategy and partly from an intercross breeding strategy (Figure 1). Phenotypic screening was applied mostly to G3 mice, although G1 mice were occasionally screened as well (see Methods). Dominant and semidominant phenotypes were detected in both G1 mice (which harbor heterozygous mutations) and G3 mice (which harbor heterozygous and homozygous mutations); some dominant and semidominant mutations were detected in the homozygous state. Screens included casual inspection for dysmorphologies affecting limbs, tail, eyes, teeth, or other aspects of body form; coat color and/or coat quality anomalies, abnormal body size (runting or obesity), and neurobehavioral anomalies. In addition, most G3 mice were subjected to one or more immunological screens, which tested the integrity of Toll-like receptor (TLR) signaling and inflammasome signaling in thioglycolate-elicited macrophages; competence to resist infection by mouse cytomegalovirus (MCMV), Rift Valley Fever Virus (RVFV), and influenza virus *in vivo*; ability of macrophages to resist infection by adenovirus and/or MCMV *ex vivo*; ability to mount a humoral response to antigenic challenges; ability to resist colitis induced by low doses of dextran sodium sulfate (DSS); and ability to mount CD8 T cell and/or NK cell dependent responses; and monitored blood cells for anomalies of hematopoiesis and/or immune cell development.

### The Mutagenetix database

Mutagenetix is an online database of the ENU-induced mouse mutations generated in the Beutler laboratory (Center for the Genetics of Host Defense, UT Southwestern Medical Center). Also described on Mutagenetix are ENU-induced mutations generated in the Goodnow laboratory (The John Curtin School of Medical Research, Australian National University); these are excluded from analysis and discussion in this paper. For each phenotypic mutation, the position and phenotypic effect of the mutation is given. In addition, detailed information about the mutated gene, the localization, structure and function of the encoded protein, and the putative mechanism of the mutation is provided, along with links to the applicable literature resources; this information distinguishes Mutagenetix from other mutation databases. A list of ENU-induced incidental mutations is also available. The phenotypic and incidental mutations can be sorted by gene name, chromosome, mode of inheritance (or zygosity in the case of incidental mutations), type of mutation, and the predicted effect of the mutation as determined by PolyPhen-2 [15]. Mutagenetix also provides up-to-date statistics of the mutation types, ENU-induced DNA changes, and amino acid changes found in the database.

Mutagenetix is intended for the sharing of discoveries and of mutant stocks with the scientific community. It provides information and links for obtaining most mutant stocks, which are deposited and made available from public repositories. It may also be useful as a template for other databases of ENU-induced mutations. Mutagenetix is open access and available at: http://mutagenetix.utsouthwestern.edu[16]. The Beutler laboratory also uses LabArchives for storage of data. An archived version of the database as of this writing is available at http://dx.doi.org/10.6070/H4VD6WC9 in a LabArchives Notebook [17].

To date, 190 phenovariants, detected among G1 and/or G3 mice as a result of observation and the above-mentioned phenotypic screens, have been publicly described on Mutagenetix. Each instance of phenovariance was caused by a single distinct mutation. 185 instances of phenovariance were ascribed to specific mutations. These mutations affected 129 genes. Multiple alleles were observed in the case of 43 genes, as summarized in Table 1. All of the phenotypes and their accompanying mutations are described in detail at http://mutagenetix.utsouthwestern.edu [16], and in the LabArchives notebook (http://dx.doi.org/10.6070/H4VD6WC9) [17].

**Table 1:** Genes with multiple ENU-induced alleles

**Type of mutation**	**Gene symbol**	**Chromosome**	**ENU-induced alleles**
**Phenotypic Mutations**	*Tlr9*	9	8
	*Slc45a2*	15	7
	*Adamts20*	15	4
	*Lepr*	4	4
	*Lyst*	13	4
	*Myo5a*	9	4
	*Nlrp3*	11	4
	*Oca2*	7	4
	*Tlr6*	5	4
	*Atp7a*	X	3
	*Krt33a*	11	3
	*Myd88*	9	3
	*Tmprss6*	15	3
	*Tyr*	7	3
	*Agtpbp1*	13	2
	*Atp11c*	X	2
	*Dock2*	11	2
	*Edaradd*	13	2
	*Hps5*	7	2
	*Hr*	14	2
	*Kcnj8*	6	2
	*Kit*	5	2
	*Krt25*	11	2
	*Muc2*	7	2
	*Ptpn6*	6	2
	*Rag1*	2	2
	*Rorb*	19	2
	*Stat1*	1	2
	*Tlr4*	4	2
	*Tlr7*	X	2
	*Zap70*	1	2
**Incidental Mutations**	*A430110N23Rik*	7	2
	*Chd2*	7	2
	*Dido1*	2	2
	*Fat1*	8	2
	*Ftsj3*	11	2
	*Gm8251*	1	2
	*Gpr98*	13	2
	*Nckap5*	1	2
	*Nf1*	11	2
	*Pde6b*	5	2
	*Samd4*	14	2
	*Ttll5*	12	2

### Phenotypic vs. incidental mutations

The mutational cause of some phenotypes was determined through a candidate approach, based on similar effects caused by other mutations in known genes. In other cases, mapping was performed using either C3H/HeN or C57BL/10J strains as mapping partners. Candidate genes within critical regions were examined by DNA sequencing to identify the causative mutation. This process originally depended upon computer-assisted primer design carried out one exon at a time using the program Generunner (http://www.generunner.net/) (2001 through 2004). Later (2005 through 2008), a semi-automated process involving the program Prime and a Biomek FX robot was implemented for primer design [12]. Still later (2008 through 2011), whole genome sequencing was performed using the ABI/LifeTechnologies SOLiD system to detect mutations in the critical region and elsewhere in the genome [18]. These mutations were validated by capillary sequencing.

In this paper, the term “phenotypic mutations” refers to those mutations known to cause phenotype, as determined by stringent methods including: identification of a mutation within a gene for which other alleles are known to cause an identical phenotype; positional restriction of the phenotype to a critical region; genetic complementation analysis; phenotypic rescue by BAC transgenesis; and/or gene knockout. The term “incidental mutations” refers to those mutations that are not known to cause phenotype, but were found in the course of whole genome sequencing, often performed in search of the cause of a phenotype. Incidental mutations may indeed cause phenotype, but were in no case responsible for the phenotype of immediate interest that led to the performance of whole genome sequencing. Mutations within coding region and within the ten proximal or distal bases of each intron were counted as incidental mutations; deeper intronic mutations or intergenic mutations were not counted in the analysis.

We also use the term “overt null alleles” to describe frameshift, nonsense, and critical splicing mutations as these have a high likelihood of affecting protein function. We note that only nonsense and critical splicing alleles are readily detectable by whole genome or whole exome sequencing.

### Types of alleles observed among phenotypic mutations

43 of the 185 phenotypes were autosomal dominant or semidominant, 2 were X-linked dominant, 124 were autosomal recessive, 8 were X-linked recessive, and 8 were not fully characterized with regard to inheritance pattern (Table 2).

**Table 2:** Mutation types generated by ENU

	**Phenotypic**							**Incidental**			
	**Autosomal recessive**	**X-linked recessive**	**Autosomal dominant (or semidominant)**	**X-linked dominant**	**Unknown**	**Total**	**% of total**	**Homozygotes**	**Heterozygotes**	**Total**	**% of total**
**Missense**	63	6	35	1	6	111	62.1	130	200	330	82.0
**Noncritical splice donor site**	4	0	0	0	1	5	2.8	9	13	22	5.5
**Noncritical splice acceptor site**	5	0	1	0	0	6	3.4	13	9	22	5.5
**Nonsense**^**†**^	27	1	5	1	1	35	19.8	7	12	19	4.7
**Critical splice donor site**^**†**^	17	0	0	0	0	17	26.6	4	4	8	2.0
**Critical splice acceptor site**^**†**^	3	0	1	0	0	4	2.2	0	1	1	0.3
**Total**	119*	**7**	42**	**1**	**8**	**178**		**163**	**239**	**402**	

†Mutations considered to be overt nulls.

*Large deletions (3 alleles), small insertions (2 alleles), and a dinucleotide substitution (1 allele) were not included in the total.

**A small deletion (1 allele) was not included in the total.

All phenotypes ascribed to mutations to date resulted from nucleotide changes that alter coding sense. Among the 185 phenotypic mutations just mentioned, 3 were large deletions and 1 was a dinucleotide substitution. Among the 181 remaining mutations (presumed to have been caused by ENU and affecting single bases), 35 were nonsense alleles, 3 were frameshift alleles (1 single base deletion and 2 single base insertions), 21 were critical splice junction defects altering either of the two nucleotides at the proximal or distal ends of introns (17 affecting donor sites and 4 affecting acceptor sites), and 11 were non-critical splice junction defects with documented effects on splicing (1 splice donor site created; 4 splice donor sites destroyed; 6 splice acceptor sites destroyed). The distances of the non-critical splice site mutations from the exon boundaries are shown in Table 3. 111 of the phenotypic mutations were single-base substitutions causing missense errors (Table 4).

**Table 3:** Distance of splice site mutations from exon boundary

**Type of mutation**	**Distance from exon boundary (bp)**	**Allele**	**Gene symbol**	**Nature of mutation**
Critical splice donor site	1	*aoba*	*Col4a4*	G→A
	1	*bat*	*Frem1*	T→C
	1	*bullet gray*	*Ap3b1*	G→T
	1	*mister clean*	*Hr*	G→A
	1	*tortellini*	*Tgm3*	G→A
	1	*warmflash*	*Flt3*	G→A
	1	*zuckerkuss*	*Slc45a2*	G→T
	2	*drunk*	*Agtpbp1*	T→A
	2	*feeble*	*Slc15a4*	T→A
	2	*frazz*	*Dock2*	T→A
	2	*frog*	*Epha4*	T→C
	2	*iron-man*	*Trfr2*	T→C
	2	*seal*	*Col1a1*	T→A
	2	*souris*	*Lyst*	T→A
	2	*styx*	*Inpp5d*	T→A
	2	*toffee*	*Hps5*	T→C
	2	*wobbley*	*Atcay*	T→A
Non-critical splice donor site	3	*salt and pepper*	*Dtnbp1*	A→T
	5	*nut*	*Myo5a*	G→A
	5	*odd*	*Lepr*	G→T
	6	*atchoum*	*Eif2ak4*	T→C
Critical splice acceptor site	2	*Joker*	*Itgb2*	A→T
	2	*mask*	*Tmprss6*	A→G
	2	*rio*	*Agtpbp1*	A→G
	2	*torpid*	*Tirap*	A→T
Non-critical splice acceptor site	5	*Sluggish*	*Map3k8*	T→A
	7	*Minnie*	*Muted*	T→A
	8	*splotch2*	*Adamts20*	T→A
	11	*frizz*	*Dock2*	T→A
	13	*poison*	*Stat1*	T→A
	17	*koala*	*Mlph*	A→G
Splice donor site created	57	*jinx*	*Unc13d*	C→A

**Table 4:** Single-base ENU-induced missense mutations

**Allele**	**Gene symbol**	**Nature of mutation**	**Amino acid substitution**	**Allele**	**Gene symbol**	**Nature of mutation**	**Amino acid substitution**
***3d***	*Unc93b1*	A→G	H412R	***Meager***	*Tlr9*	A→T	N210Y
***4-limb clasper***	*Rorb*	T→C	S87P	***Muskatenwein***	*Muc2*	T→A	I2172N
***achtung***	*Edaradd*	A→T	I105F	***Myd88rev1***	*Myd88*	T→A	F285I
***achtung2***	*Edar*	C→A	P349Q	***ND1***	*Nlrp3*	T→G	C987W
***add***	*Tomt*	G→T	R48L	***ND5***	*Nlrp3*	T→C	W956R
***allia***	*Cd247*	A→T	D36V	***ND6***	*Nlrp3*	A→G	R586G
***artemis***	*Bicc1*	C→T	T86I	***ND7***	*Nlrp3*	T→C	C816R
***Bemr3***	*Rho*	T→C	C185R	***otiose***	*Irak4*	T→C	I327T
***bronze***	*Pah*	T→C	L242P	***pale rider***	*Tyr*	T→A	V427D
***brown***	*Atp7a*	T→C	I483T	***PanR1***	*Tnf*	C→A	P217T
***cartoon***	*Mmp14*	T→C	S466P	***panr2***	*Ikbkg*	T→C	L153P
***Casper***	*Kit*	A→G	D676G	***phoebus***	*Aqp3*	T→C	V43A
***charbon***	*Oca2*	A→G	T382A	***pinkie***	*Rxra*	T→A	I273N
***CpG1***	*Tlr9*	T→C	L499P	***Plush***	*Krt25*	T→A	L86Q
***CpG11***	*Tlr9*	T→C	S358P	***pococurante***	*Myd88*	T→A	I179N
***CpG2***	*Tlr9*	A→T	Q985L	***Polished***	*Krt33a*	T→G	Y232D
***CpG3***	*Tlr9*	T→A	V214E	***Polished2***	*Krt33a*	T→A	C100R
***CpG5***	*Tlr9*	T→C	L393P	***Possum***	*Scn10a*	A→G	T790A
***CpG6***	*Tlr9*	G→A	G1028R	***Pretty2***	*Kit*	A→T	I787F
***CpG7***	*Tlr9*	G→T	R613L	***prune***	*Hr*	T→G	Y1082D
***crab***	*Cd79a*	T→A	C50S	***queen***	*Plcg2*	T→A	I346R
***crusty***	*Foxp3*	T→A	I350N	***quicksilver***	*Oca2*	G→A	E453K
***csp***	*Ltbp3*	T→C	C452R	***Rough-fur***	*Krt10*	A→T	E172V
***Dalmatian***	*Sox10*	T→G	N131K	***rsq1***	*Tlr7*	C→T	T68I
***deer***	*Mc1r*	A→G	Y150C	***rsq2***	*Tlr7*	A→T	N182Y
***domino***	*Stat1*	T→A	V319E	***scanT***	*Zbtb1*	T→C	C74R
***eel***	*Npr3*	A→T	I384F	***Schlendrian***	*Muc2*	G→T	C561F
***elektra***	*Slfn2*	T→A	I135N	***siamese***	*Tyr*	A→G	H420R
***Endeka***	*Irf1*	A→G	D47G	***sinecure***	*Rhbdf2*	A->T	I387F
***F4/80***	*Emr1*	A→G	Y579C	***silver decerebrate***	*Myo5a*	G→T	S693I
***flake***	*Scd1*	C→A	T227K	***Sinuous***	*Krt25*	T→C	S88P
***ghost***	*Tyr*	A→G	H363R	***snowflake***	*Oca2*	C→T	P459L
***gimpy***	*Wnt7a*	T→A	C339S	***sos***	*Kcnj8*	T→C	I318T
***goofy***	*Alx4*	A→G	H272R	***Southbeach***	*Mc4r*	T→C	L300P
***grasshopper***	*Rorb*	T→C	M1T	***spelling***	*Atp11c*	T→A	I355K
***grey goose***	*Slc45a2*	T→C	L180P	***sphinx***	*Gimap5*	G→T	G38C
***honey***	*Irf4*	A→T	R96S	***Spikey***	*Krt33a*	T→A	I353N
***iconoclast***	*Lck*	T→C	L300P	***spin***	*Ptpn6*	T→A	Y208N
***inept***	*Irf7*	A→G	D110G	***spin2***	*Ptpn6*	T→C	L86P
***insouciant***	*Tlr6*	T→C	V327A	***stamper-coat***	*Hps6*	T→A	W71R
***iron10***	*Cp*	T→C	L1033P	***sweater***	*Slc45a2*	A→C	H233P
***jitter***	*Kcnn2*	T→C	L168P	***Tigrou-like***	*Atp7a*	C→T	A998V
***june gloom***	*Slc45a2*	T→C	S378P	***trebia***	*Zap70*	A→T	Y492F
***king***	*Card11*	T→A	L525Q	***TremorD***	*Scn8a*	G→T	W935L
***L1***	*Gja8*	T→C	S50P	***Unnatural***	*Klrb1c*	A→G	D100G
***L10***	*Crygb*	A→T	I4F	***Untied***	*Prkcb*	T→C	Y417H
***L1N***	*Cryaa*	T→G	Y141D	***Velvet***	*Egfr*	A→G	D857G
***L23***	*Crygd*	T→A	V76D	***walla***	*Cd40lg*	T→C	S221P
***lackadaisical***	*Myd88*	A→G	Y116C	***wanna***	*Zap70*	A→G	H58R
***languid***	*Tlr2*	A→T	N487I	***wavedX***	*Adam17*	T→A	F343I
***lps3***	*Tlr4*	A→T	D709V	***whitemouse***	*Oca2*	T→A	W725R
***Lps4***	*Tlr4*	G→A	G724D	***woodrat***	*Mbtps1*	A→G	Y496C
***m2sd3***	*Tlr6*	T→C	I441T	***woolly***	*Sgk3*	T→C	C346R
***macro-1***	*Ifnar1*	A→C	T341P	***yuki***	*Slc45a2*	T→G	S92R
***macro-2***	*Ifnar1*	A→G	M1V	***zeitgeist***	*Med30*	A→T	I44F
***madcow***	*Cars2*	T→A	S305T	***zigzag***	*Lfng*	T→C	V204A

In all, 59 ENU-induced phenotypes were caused by readily detectable overt null alleles, as against 122 ENU-induced phenotypes that were caused by other types of allele (a ratio of 1 to 2.07, or 32.57% of all phenotypic mutations). In our study as in others [4,7,19-21], the most common cause of phenotypic change induced by ENU is missense mutation.

### Types of alleles observed among incidental mutations

A total of 402 incidental mutations affecting 390 genes were observed and validated by capillary sequencing. These mutations were derived from whole genome sequencing of 37 strains at G3 level or in subsequent generations, and one strain at the G1 level (in which 11 mutations were validated).

A total of 19 nonsense alleles, 1 critical splice acceptor allele, and 8 critical splice donor alleles were detected for a total of 28 overt null alleles. 330 missense alleles, 22 non-critical splice donor alleles, and 22 non-critical splice acceptor alleles were also recorded. Of all 402 mutations, 163 were detected in the homozygous or hemizygous state, whereas 239 were detected in the heterozygous state (Table 2).

The ratio of overt null alleles to other alleles is smaller among incidental mutations than among phenotypic mutations: 1 to 13.4, or 6.94% of the mutation total. However, the frequency of specific individual base substitutions (sense strand) did not differ significantly on comparison of phenotypic vs. incidental mutations.

### A global estimate of how commonly ENU-induced missense mutations cause phenovariance

By definition, 100% of the missense alleles in the phenotypic set are detectable by phenotypic screening. The requirement that they cause phenotype enriches overt null alleles among phenotypic mutations compared to incidental mutations, and would similarly be expected to enrich deleterious missense alleles. Moreover, the degree of enrichment of deleterious missense alleles, whether overtly null, covertly null, or functionally hypomorphic to the extent that they cause phenotype, should be equivalent to that of overt null alleles. As described above, overt null alleles accounted for 32.57% of all ENU-induced phenotypic mutations, whereas they comprised only 6.94% of ENU-induced incidental mutations, an enrichment of 4.70-fold in the phenotypic class relative to the incidental class. We infer that deleterious (*i.e.* phenotypically detectable) missense alleles should also be 4.70 times more common among phenotypic mutations as they are among incidental mutations. To a first approximation, we therefore infer that 21.3% of the missense alleles induced by ENU and observed among incidental mutations would be phenotypically detectable if a suitable assay for phenotype, equivalent in sensitivity to the visual inspection and immunological assays we used, were applied.

### The most and least deleterious types of ENU-induced amino acid substitutions

Based on inspection of the codon table, a total of 182 types of coding changes can result from single nucleotide substitutions, inclusive of the elimination of start codons and stop codons. Not all of these changes can necessarily be achieved by ENU mutagenesis. We compiled a list of all non-splicing errors from both phenotypic and incidental mutation lists. Among phenotypic mutations, 145 coding changes of 61 types were recorded. Among incidental mutations, 349 coding changes of 103 types were recorded. Both groups together accounted for 494 coding changes of 114 types.

**Figure 2:**
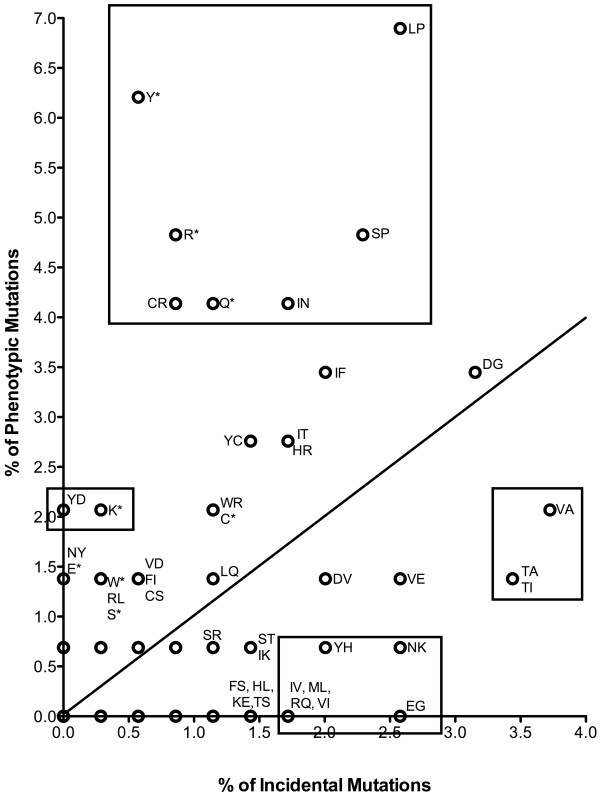
**Frequency of amino acid changes among phenotypic and incidental mutation classes.** The line represents an equal frequency between phenotypic and incidental mutations. Boxed amino acid changes represent those that occurred significantly more frequently in the phenotypic vs. incidental mutation set, or *vice versa* .

Certain amino acid changes were observed more frequently within the phenotypic mutation set than within the incidental mutation set. We take this to imply that certain amino acid substitutions are more likely to be deleterious. These changes include S→P, L→P, I→N, C→R, and Y→D. Also overrepresented among the phenotypic mutations as compared with incidental mutations were all of the observed nonsense substitutions Y→*, R→*, K→*, and Q→*, which are expected to be strongly deleterious in most instances. On the other hand, certain substitutions appear comparatively benign, as they were found more commonly among incidental mutations than among phenotypic mutations: T→I, T→A, V→A, Y→H, N→K, and E→G, and several others. A graphical comparison of phenotypic and incidental mutations is displayed in Figure 2.

### Computational prediction of mutation effects: sensitivity and specificity

**Figure 3:**
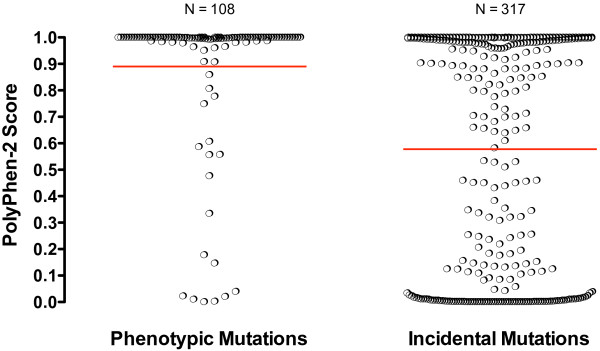
**PolyPhen-2 scores of phenotypic and incidental mutations.** Red lines indicate means.

Computational algorithms such as Polymorphism Phenotype (PolyPhen-2), MUpro, and Sort Intolerant from Tolerant (SIFT) can be used to assess the likelihood of loss of function when a given substitution is introduced into a protein. PolyPhen-2 surveys the nature of the amino acid change, secondary structure, and conservation among species to estimate the likelihood of damage; splice site mutations and premature stops are not evaluated. We used PolyPhen-2 to assess all phenotypic and incidental missense mutations in our dataset. Comparison of the PolyPhen-2 predictions for phenotypic *versus* incidental mutations demonstrated that PolyPhen-2 is quite sensitive in detecting damage potential. Of 110 missense mutations known to cause phenotype, all but 22 (80.0%) had scores equal to or exceeding 0.95 (the lower cutoff for declaring a mutation “probably damaging”) (Figure 3, left). The mean score was 0.890. For 3 mutations, no information was returned by PolyPhen-2.

The scores assigned to 330 incidental missense mutations contrasted strikingly with those of phenotypic mutations in distribution. Nearly half of all incidental mutation scores were beneath 0.5 (mean score 0.578), and only 125 (37.9%) had scores equal to or exceeding 0.95 (Figure 3, right). For 13 mutations no information was returned by PolyPhen-2.

The specificity of PolyPhen-2 is more difficult to assess, since there is no assurance that incidental mutations do not cause phenotype. However, we have estimated that approximately 21.3% of ENU-induced missense mutations are likely to cause phenotype, which would predict that 67 of 317 incidental mutations with assignable scores should be damaging. As the actual number of incidental mutations with PolyPhen-2 scores exceeding 0.95 was 125, we therefore estimate that PolyPhen-2 is at most 54% specific in declaring mutations sufficiently deleterious to cause phenotype.

### Strand asymmetry of ENU-induced mutations in both phenotypic and incidental mutations

As previously reported [2,4,5], the most common nucleotide substitutions caused by ENU are A→T transversions and A→G transitions. Interestingly, however, the corresponding T→A transversions and T→C transitions are far more common in the sense strand, indicating strand asymmetry in mutagenesis. In order to judge the significance of strand asymmetry, we counted all nucleotide substitutions predicted to cause a change in coding sense, taking as our target the longest coding transcript of every protein-encoding gene listed in the NCBI database. We additionally counted all nucleotide substitutions within critical splice junctions, assuming that these mutations, too, would cause a change in coding sense. Our intention was to evaluate the relative opportunity for each missense, nonsense, or critical splice junction mutation to occur, within either the sense strand or the antisense strand of the coding region. We then made statistical comparisons between observed base changes in each strand based on exact binomial probability (Table 5).

**Table 5:** Frequencies of ENU-induced DNA base changes in the sense strand

**DNA base change**	**p**^**†**^	**Phenotypic mutations**		**Incidental mutations**		**Phenotypic + Incidental mutations**	
		**Mutation number**	P values*	**Mutation number**	P values*	**Total number**	P values*
**A→T**	0.5470	23	0.00397	39	0.000435	62	0.00000725
**T→A**	0.4530	39		64		103	
**A→G**	0.5780	20	0.00071	70	0.9425	90	0.00322
**T→C**	0.4220	36		66		102	
**A→C**	0.5308	2	0.06310	8	0.3982	10	0.09730
**T→G**	0.4692	7		9		16	
**G→T**	0.5341	10	0.6600	14	0.3876	24	0.4289
**C→A**	0.4659	8		15		23	
**G→A**	0.5563	9	0.1202	35	0.1401	44	0.0533
**C→T**	0.4437	13		37		50	
**G→C**	0.5187	0	--	1	1.0	1	1.0
**C→G**	0.4813	0		0		0	
**Total**		167**		**358**^**††**^		**527**	

^†^Relative probability of non-synonymous nucleotide change in the sense strand (see Methods).

*Likelihood of observed or greater departure from the expected ratio by exact binomial calculation.

**Not included were 11 non-critical splice junction mutations, 3 large deletions, 2 single base insertions, 1 single base deletion, and 1 dinucleotide transition.

^††^Not included were 44 non-critical splice junction mutations.

The null hypothesis holds that both sense and antisense T residues that yield a coding change when mutated to an A are equally valid targets for mutagenesis. This hypothesis would predict that T→A mutation in the sense strand should occur 82.817% as often as T→A mutation in the antisense strand. However, with a P value of 0.00397, the hypothesis is rejected for phenotypic mutations; with a P value of 0.000435 it is rejected for incidental mutations; and with a P value of 0.00000725 it is rejected for both phenotypic and incidental mutations. T→A substitution actually occurs 1.7 times more frequently in the sense strand as in the antisense strand.

Interestingly T→C mutations show even stronger strand asymmetry among phenotypic mutations, and are over-represented to a highly significant degree in the sense strand (P = 0.00071), but are not over-represented among incidental mutations. This observation is consistent with the interpretation that T→C mutations in the sense strand tend to cause more destructive effects than A→G mutations (which correspond to T→C mutations in the antisense strand). T→C substitutions in the sense strand disproportionately affect valine, leucine, cysteine, serine, methionine, and all of the aromatic amino acids as compared to A→G. A selective trend toward significance among phenotypic mutations is also observed for A→C/T→G (sense/antisense) among phenotypic mutations, and for G→A/C→T (sense/antisense) among both phenotypic and incidental mutations (Table 5).

These findings are consistent with the earlier observations of Takahasi *et al.*, who posited that transcription-coupled repair mechanisms might account for strand asymmetry [21]. However, it is possible that selection bias based on the nature of coding change may also contribute to the effect measured in phenotypic mutations.

## Discussion

We have compared two classes of ENU-induced mutations: ‘phenotypic’ mutations (mutations identified as causative for particular phenotypes) and ‘incidental’ mutations (mutations that are not known to cause phenotype). Phenotypic and incidental mutations differed in several respects, permitting us to make several conclusions regarding the creation of phenotype by ENU. First, the frequency of different types of alleles (*i.e.* missense, nonsense, splice donor site, *etc.*) observed among phenotypic mutations in both G1 and G3 mice differed significantly from those of incidental mutations. In particular, overt null alleles, considered to include frameshift, nonsense, and critical splicing mutations, were far more frequent (~4.7x increased) among phenotypic mutations than incidental mutations. This enrichment for deleterious alleles reflects the process of selection that identifies mutations of the phenotypic class. By considering that the same selective process also enriches deleterious alleles of other types, we calculated that globally, ENU-induced missense mutations (the most common type of ENU-induced mutation) may be expected to cause detectable phenovariance at a frequency of approximately 21.3%.

The efficiency of ENU mutagenesis in the creation of phenotype in mice is thus relatively low, albeit compensated by the abundance of mutations that are transmitted to each G1 mouse. It must be emphasized, of course, that declaration of “phenotype” is entirely dependent on assay sensitivity and precision. The data we have presented are derived from a number of immunological assays and from relatively casual inspection of animals for visible or behavioral abnormalities. In the case of TLR signaling screens, assay variance (measurement of TNF secretion by peritoneal macrophages) typically encompasses a 10 fold range from lowest to highest value. If, in an imaginary scenario, the phenotype at issue were instead defined by measurement of a particular enzymatic activity, and if the relevant assay could be performed with precision sufficient to reduce variance to one percent of the mean, a greater fraction of missense mutations might be scored as phenovariants.

In examining phenotypic *versus* incidental mutations, we also observed that certain amino acid substitutions were more likely to be found among either the phenotypic or the incidental class of mutations. For example, S→P, L→P, I→N, C→R, Y→D, Y→*, Q→*, K→*, and R→* were more common among phenotypic mutations than incidental mutations, strongly suggesting that, irrespective of location within the polypeptide chain, these specific substitutions are more likely to be damaging to protein function than not. In many cases, these substitutions are predicted to result in major changes to the biochemical properties of the protein locally, such as polar to nonpolar (S→P), nonpolar to basic (C→R), or nonpolar to polar (I→N). Substitutions to proline (as in S→P and L→P) also often conformationally constrain secondary structure due to the cyclic structure of proline. Conversely, the substitutions T→I, T→A, V→A, V→E, N→K, and E→G occurred more frequently among incidental mutations than phenotypic mutations. These data suggest that in general, certain amino acid substitutions are detrimental and others are innocuous. Because ENU tends to affect some nucleotides preferentially and exhibits strand specificity, noted earlier by others [2,4,5,21] and confirmed here by our own studies, its tendency to generate changes that cause phenotype is best accomplished empirically, as we have done here.

As an extension of the analysis of specific amino acid substitutions, we also evaluated phenotypic and incidental missense mutations using PolyPhen-2, which computationally predicts the impact of sequence variation on protein function. PolyPhen-2 has been reported to achieve accurate positive prediction rates of 92% and 73% using the HumDiv- and HumVar- trained program versions, respectively [15]. In our analysis we used the HumDiv-trained PolyPhen-2, as recommended for evaluation of rare alleles at loci that may potentially be involved in complex phenotypes. In agreement with Adzhubei *et al.* we found that among phenotypic mutations, all of which by definition are damaging, 80% scored at 0.95 or higher and were thus correctly labeled as “probably damaging.”

PolyPhen-2 was at best about 54% specific in labeling incidental mutations as “probably damaging.” We note, however, that the creation of a phenotype may often require more than causing damage, however slight, to protein function; hence, “causing phenovariance” may be viewed as a more stringent criterion than “causing damage.” For this reason, the apparent low specificity of PolyPhen-2 is not unexpected.

## Conclusion

We estimated that 21.3% of ENU-induced missense mutations cause phenotypes detectable in typical screening assays. We also identified those ENU-induced amino acid substitutions that are most likely—and those that are least likely—to cause phenotypic change. Knowledge of the types of mutations caused by ENU and their relative likelihood of causing phenotype may be incorporated into estimates of genome saturation when a screen has been performed on a specific population of G3 mice. Such an estimate is best made by simulating mutagenesis in silico. Because the coding region of the mouse has been defined and well annotated, the characteristics of ENU-induced mutations have been relatively well studied, and the frequency of ENU-induced mutations has been estimated, simulations can closely approximate mutagenesis as it is actually performed. While it is safe to assume that overt null alleles observed in simulation would indeed be deleterious, it has been more difficult to determine the likelihood that individual missense mutations will lead to perceptible phenotypic change. Our data provide a reasonable basis for making such inferences, and for estimating the fraction of genes that have been mutated to a state of phenovariance.

## Methods

Links to the full protocols can be found in Table 6.

**Table 6:** Protocols and screens used to identify ENU-induced mutations

**Protocol or screen**	**On-line resource**
**Mutagenizing male mice with ENU**	http://dx.doi.org/10.6070/H4QN64NZ
**Genetic Mapping: Whole Genome Mapping and Fine Mapping**	http://dx.doi.org/10.6070/H4G44N67
**Genetic Mapping by Bulk Segregation Analysis**	http://dx.doi.org/10.6070/H4KW5CX7
***Ex Vivo *Macrophage Screen for Control of Viral Infection**	http://dx.doi.org/10.6070/H4Z60KZS
***In Vivo *Rift Valley Fever Virus (RVFV) Susceptibility Screen**	http://dx.doi.org/10.6070/H4JW8BSV
**Toll-like Receptor (TLR) Signaling Screen**	http://dx.doi.org/10.6070/H4NP22CR
**NALP3 Inflammasome Screen**	http://dx.doi.org/10.6070/H4BG2KWT
**MCMV Susceptibility and Resistance Screen**	http://dx.doi.org/10.6070/H41Z429S
**T-dependent Humoral Response Screen**	http://dx.doi.org/10.6070/H4X63JTD
**T-independent B Cell Response Screen**	http://dx.doi.org/10.6070/H4SF2T33
**DSS-induced Colitis Screen**	http://dx.doi.org/10.6070/H42Z13F5
***In Vivo *NK Cell and CD8+ T Cell Cytotoxicity Screen**	http://dx.doi.org/10.6070/H4PN93HK

### Mice

Mice were housed and bred under specific pathogen free conditions in polycarbonate cages with corn cob bedding in The Scripps Research Institute vivarium. Mice were housed at four adults per cage in self-watering cage racks kept in 25°C rooms with a 12 h light/12 h dark light cycle. Mice had unlimited access to rodent chow and water. C57BL/6J and C57BL/10J mice were purchased from The Jackson Laboratory. All studies were performed in accordance with the guidelines established by the Institutional Animal Care and Use Committee of The Scripps Research Institute.

### ENU Mutagenesis and genetic mapping of mutations

ENU was prepared fresh for each set of injections at 100 mg/ml, as previously described [20]. The precise concentration of ENU was determined by absorbance at λ = 398 nm and calculated based on the formula 0.72 O.D. = 1 mg/ml of ENU. After ENU preparation, C57BL/6J male mice were anesthetized with a xylazine/ketamine injection (100 μl i.m.) and injected i.p. with 100 mg ENU/kg of body weight once per week for three weeks. After the last injection, the mice were housed one per cage for 12 weeks to allow for recovery of fertility.

To map a mutation, the mutant stock (C57BL/6J background) was outcrossed to a second strain (C57BL/10J or C3H/HeN) and then F1 hybrids were either intercrossed or backcrossed to the mutant stock (for recessive mutations) or mated to the outcross strain (for dominant mutations) [20]. Offspring of F1 mice were subsequently analyzed phenotypically (see below for descriptions of the phenotypic screens used) and a critical region was established by genetic linkage mapping using approximately 120 polymorphic markers across the whole genome and additional markers close to the mutation as necessary. Bulk segregation analysis (BSA) was used to map some mutations, as described [22]. Incidental mutation data were obtained by whole genome sequencing using versions 2–4 of SOLiD technology (Life Technologies, Grand Island, NY).

### Phenotypic screens

In total, approximately 100,000 G3 mice from 8,000 pedigrees were subjected to screening. Mice were allocated to screens such that some mice were used in only one screen, whereas others were tested serially in several screens; non-invasive screening was performed preceding invasive screening. Mice were subjected to two screens at most, with a one week rest period between screens. Statistical analysis of data from each screen was performed as described in references cited on the relevant mutation page in Mutagenetix or the LabArchives Notebook.

#### MCMV susceptibility and resistance screen

MCMV (Smith strain) was propagated in BALB/c mice and harvested from salivary glands. To screen for susceptibility or resistance of G3 mice to MCMV [23], approximately 22,600 female mice were inoculated intraperitoneally with either 1x10^5^ pfu MCMV, an inoculum normally harmless to C57BL/6J mice, or 1x10^6^ pfu MCMV, a dose that typically causes death of C57BL/6J mice by five days post-infection. Mice were observed for sickness or death for one week following infection. Putative susceptible mutants were those that sickened or died in response to infection with 1x10^5^ pfu MCMV; putative resistant mutants were those that survived after infection with 1x10^6^ pfu MCMV.

#### Toll-like receptor (TLR) signaling screen

Briefly, thioglycolate-elicited peritoneal exudate cells were isolated from approximately 39,000 male and female G3 mice, plated at 5x10^4^ cells/well in 96-well plates, and stimulated with the following TLR ligands at the indicated concentrations: lipopolysaccharide (LPS; 800 pg/ml), poly(I:C) (150 μg/mL), Pam_3_CSK_4_ (30 ng/mL), resiquimod (30 ng/mL), CpG oligodeoxynucleotides (15 μg/mL), peptidoglycan (1 μg/mL), MALP-2 (600 pg/mL). Cells were stimulated for 4 h at 37°C/5% CO_2_ in a humidified incubator. The concentration of tumor necrosis factor (TNF)-α in the culture medium was determined by bioassay using L-929 cells, for which TNF is cytotoxic.

#### NALP3 inflammasome screen

Peritoneal exudate cells isolated from approximately 7,500 male and female G3 mice and plated as described above were stimulated with LPS (100 ng/mL) for 4 h, followed by nigericin (10 μg/mL) for 1 h at 37°C/5% CO_2_ in a humidified incubator. The concentration of interleukin (IL)-1β in the culture medium was determined by ELISA.

#### In vivo RVFV susceptibility screen

The mutagen-attenuated recombinant strain arMP-12 [24] was used for the screen and propagated in VeroE6 cells. Approximately 900 female G3 mice were injected intraperitoneally with 1x10^5^ pfu of RVFV, which initiates an infection controlled by C57BL/6J mice with no signs of illness. The mice were observed daily for signs of illness; symptomatic mice were sacrificed and blood, liver, spleen, and brain collected. Viral titers were measured in the infected tissues by standard plaque assay using VeroE6 cells.

#### Ex vivo macrophage screen for control of viral infection

The screen was performed as described [25]. Briefly, 10^5^ thioglycolate-elicited peritoneal exudate cells isolated from approximately 7,000 male and female G3 mice were infected for 24 h with MCMV-GFP [26] at MOI 1, or for 72 h with Ad5-F16-GFP [27] at 10^4^ particles per cell. Cells were incubated at 37°C with 5% CO_2_ in a humidified incubator. The number of infected cells was determined by counting the number of GFP^+^ cells by flow cytometry. MCMV-GFP was a gift from Dr. Chris Benedict (La Jolla Institute of Allergy and Immunology, La Jolla, CA) and was propagated as described [28]. Ad5-F16-GFP was a gift from Dr. Glen Nemerow (The Scripps Research Institute, La Jolla, CA) and was propagated as described [27,29].

#### T-dependent and T-independent humoral response screen

Briefly, approximately 7,000 male G3 mice were immunized with 2 x 10^6^ IU of a recombinant, non-replicating Semliki Forest Virus vector (rSFV) encoding βGal by i.p. injection. After ten days, mice were also immunized with 50 μg of 4-hydroxy-3-nitrophenylacetyl-AminoEthylCarboxyMethyl-Ficoll (NP-Ficoll). Fourteen days after the initial immunization, blood was collected from the retro-orbital sinus and specific antibodies were measured. To detect βGal-specific IgG or NP-specific IgM, 96-well round bottom plates were coated with 2 μg/mL βGal in phosphate buffered saline or 5 μg/mL NP_23_-BSA for ELISA. Putative mutants exhibited deficient antibody responses.

#### DSS-induced colitis screen

To identify G3 mice susceptible to dextran sulfate sodium (DSS)-induced colitis, approximately 6,000 male and female G3 mice were exposed for one week to 1% (w/v) DSS in the drinking water, a concentration harmless to C57BL/6J animals. Mice were weighed daily and those displaying loss of at least 8% of their original weight by day 6 of treatment were considered putative mutants.

#### In vivo NK cell and CD8^+^ T cell cytotoxicity screen

Briefly, approximately 4,000 male and female G3 mice and control *Tap1*^*−/−*^ mice were immunized i.p. with 10^7^ mouse splenocytes expressing membrane-associated ovalbumin driven by an actin promoter (act-mOVA) [30]. Eight days after immunization, 1.33x10^5^ carboxyfluorescein succinimidyl ester (CFSE)-labeled splenocytes from syngeneic C57BL/6J mice loaded with the OVA-derived SIINFEKL peptide were injected into the tail vein of immunized mice as cytolytic T lymphocyte (CTL) target cells. Also injected were 1.33x10^5^ CSFE-labeled splenocytes from *Tap1*^*−/−*^ mice as NK cell target cells, and 1.33x10^5^ CSFE-labeled C57BL/6J splenocytes as control cells. Each target cell population was labeled with a different intensity of CSFE. Two days after injection of CSFE-labeled cells, 200 μl of blood from the orbital sinus of each mouse was extracted and the CSFE-labeled target cells were counted by flow cytometry. The percentage of NK cell and CTL targets killed was calculated as follows: % targets killed = [1-(target cells/control cells)/(ratio of target cells:control cells in *Tap1*^*−/−*^ mouse)] x 100. G3 mice that failed to kill NK or CTL target cells were considered as putative mutants.

### Statistical analysis

The statistical significance of differences in the frequency of particular nucleotide changes induced in the sense *versus* antisense strands was calculated as the exact binomial probability, where k was the number of observed mutations and p was the probability of a particular nonsynonymous coding change or critical splice junction change in the sense strand *versus* the antisense strand. As an example, to calculate p for A→G substitutions, the number of A→G sense strand substitutions predicted to cause nonsynonymous coding change or critical splice junction change was divided by the sum of A→G and T→C sense strand substitutions predicted to cause nonsynonymous coding change or critical splice junction change, given that a T→C change in the sense strand corresponds to an A→G change in the antisense strand. The number of each of the 12 possible nucleotide substitutions predicted to cause nonsynonymous coding change or critical splice junction change in the sense strand is shown in Table 7.

**Table 7:** Nucleotide changes that alter coding sense

**Mutation**	Coding sequence*	Critical Splice Junctions*	Total*
**A→C**	7557737	147033	7704770
**A→G**	6938327	147033	7085360
**A→T**	7856324	147033	8003357
**C→A**	6939729	93629	7033358
**C→G**	7387012	93629	7480641
**C→T**	5071174	93629	5164803
**G→A**	6231339	243302	6474641
**G→C**	7818308	243302	8061610
**G→T**	7818308	243302	8061610
**T→A**	6415261	212884	6628145
**T→C**	4959605	212884	5172489
**T→G**	6597408	212884	6810292
**Total**	35258130	696852	35954982

* Values are for the sense strand.

## Availability of supporting data

The data set supporting the results of this article is available in the Mutagenetix database, http://mutagenetix.utsouthwestern.edu, and in an archived version of the database in a LabArchives Notebook, http://dx.doi.org/10.6070/H4VD6WC9.

## Competing interests

The authors declare that they have no competing interests.

## Author contributions

CAN, MJB, MB, ALB, KB, BC, KC, XD, CE, PG, KH, HH, ZJ, PK, DP, SR, OMS, LS, KT, WT, SW, and NX performed phenotypic screening and genetic mapping. XL performed mouse husbandry, cryopreservation, and transgenesis. SL and YX analyzed mutation statistics. EMYM, ARM, and NGS wrote the Mutagenetix records pages describing mutations. YX analyzed DNA sequence and mapping data. BB conceived of the study and its design. EMYM, ARM, and BB compiled data, analyzed data, and wrote the manuscript. All authors read and approved the final manuscript.
